# Supplementary material for: Structure and antagonism of the receptor complex mediated by human TSLP in allergy and asthma
Source: Nat Commun. 2017 Apr 3;8:14937. doi: 10.1038/ncomms14937 (PMC5382266; doi:10.1038/ncomms14937)
Supplement: Supplementary Information — Supplementary figures, supplementary tables and supplementary references. [file ncomms14937-s1.pdf]

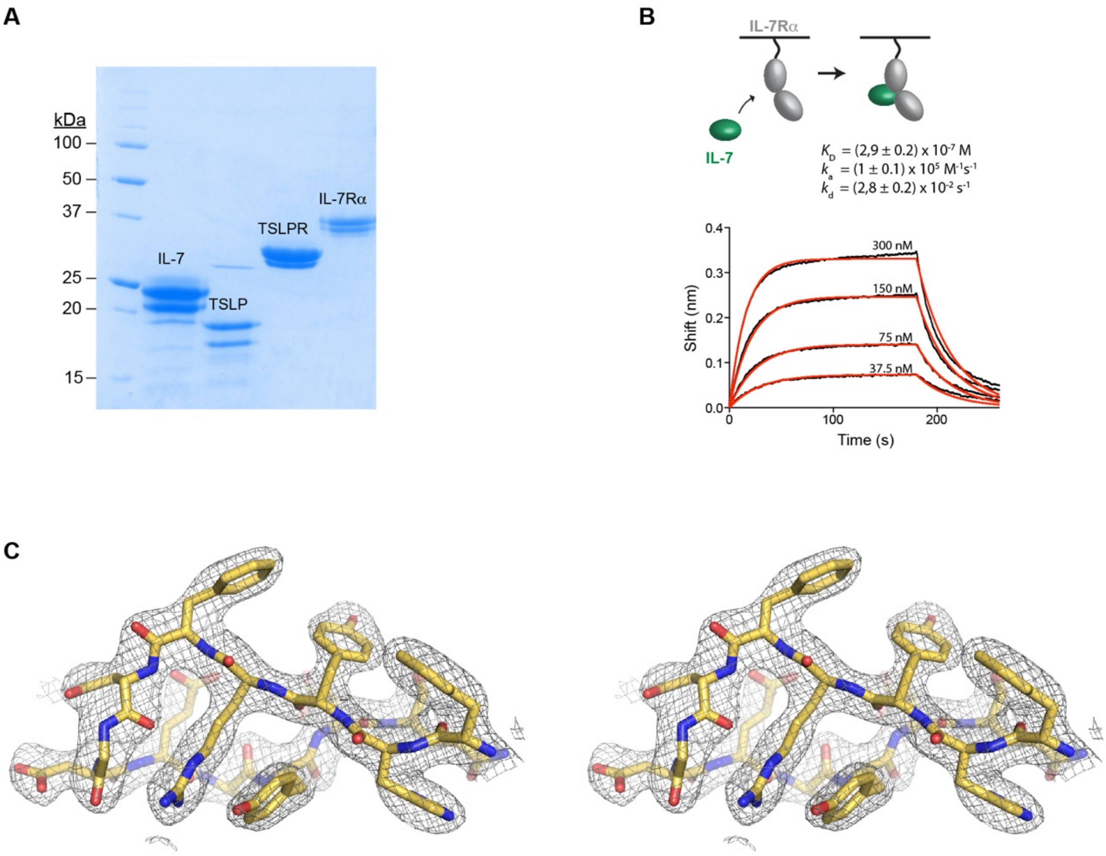

Supplementary Figure 1 (A) Coomassie-stained reducing SDS-PAGE gel of purified samples for human IL-7, $TSLP^{R127A/R130A}$ and the TSLPR and IL-7Rα ectodomains produced in stable transfected HEK293S-TetR $MGAT1^{-/-}$ cells. The theoretical protein molecular weights are 18.5 kDa for IL-7, 14.6 kDa for TSLP, 24.0 kDa for TSLPR and 25.7 kDa for IL-7Rα. Molecular weights of protein standards are indicated. (B) BLI data traces (black) and the fitted 1:1 binding model (red) are plotted as the spectral nanometer shift in function of time for the interaction of IL-7 with immobilized IL-7Rα. To generate the biosensing surface 2.5 nm of biotinylated IL-7Rα was loaded on streptavidin-coated sensor tips. The reported K_D-, k_a- and k_d-values represent average values and their standard deviations from three technical replicate experiments. (C) Stereo-view of the final Sigma-A weighted $2mF_O$-DF_C and mF_O-DF_C difference electron density maps for the TSLP:TSLPR:IL-7Rα complex around TSLPR residues 58 to 70. The $2mF_O$-DF_C map (grey) is contoured at 1 r.m.s.d. and the mF_O-DF_C map contoured at ±3 r.m.s.d. and were rendered in PyMOL[1] using a carve radius of 1.6 Å.

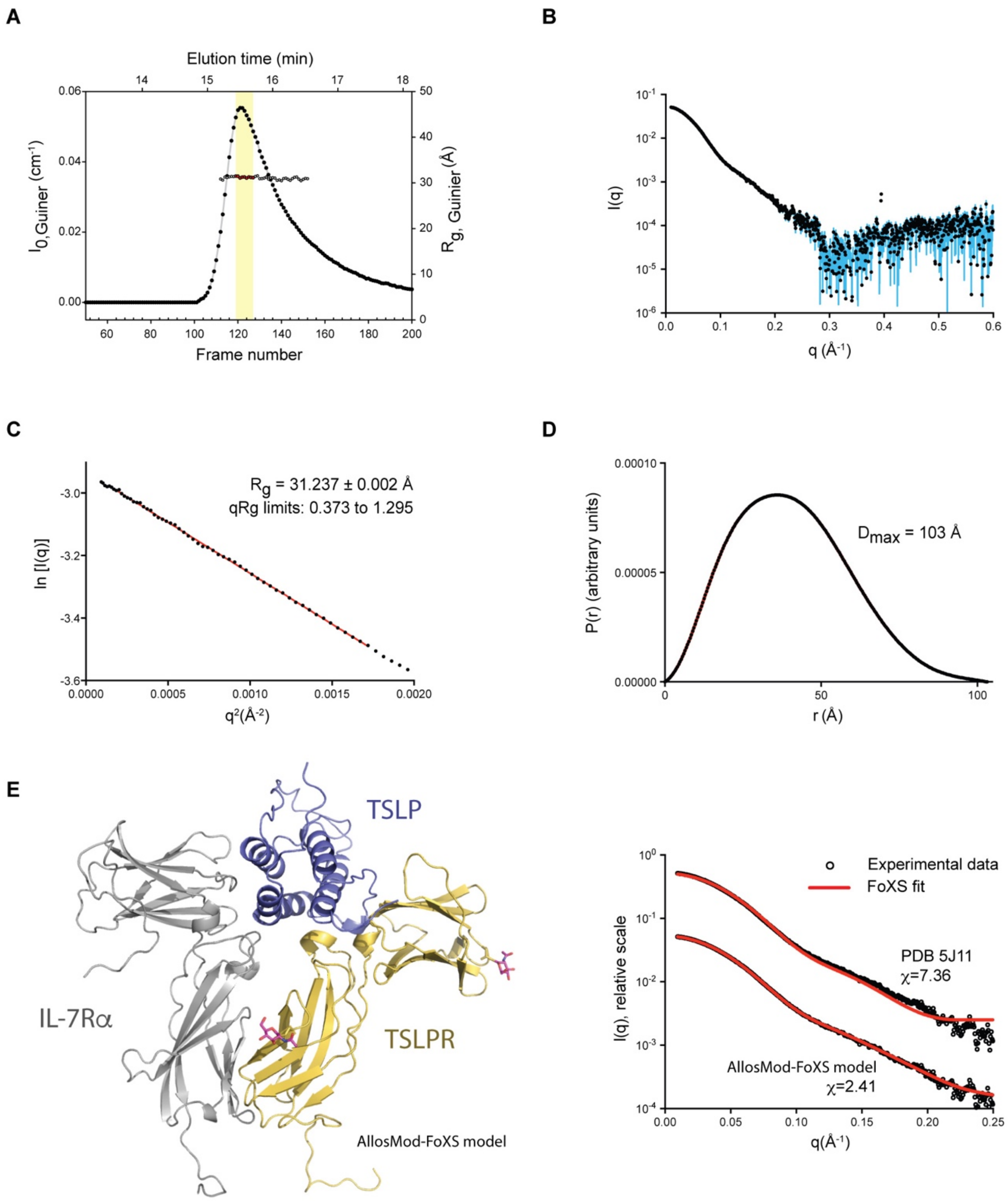

Supplementary Figure 2

In-solution SAXS studies on the glycan-minimized $TSLP^{\Delta 127-131}$:$TSLPR^{N47Q}$:IL-7Rα complex. (A) The extrapolated intensity at zero scattering angle, I_0, and radius of gyration, R_g , of the radially averaged scattering data is plotted in function of frame number and elution time. Both I_0 and R_g were obtained through Guinier analysis. For subsequent data analysis, frames 119 to 127 (shaded yellow), which have the highest scattering intensity were averaged. (B) Averaged SAXS profile plotted as the logarithm of the scattering intensity in function of momentum transfer q, with $q = (4\pi \sin\theta)/\lambda$. Standard deviations for each data point are shown as grey error bars. (C) A plot of the logarithm of the scattering intensity in function of squared momentum transfer value, q, displays linear behavior for small q-values as illustrated by linear regression (red line). The limits for the fit and reported Rg-value were determined by AutoRg. (D) Pair-distance distribution function, P(r), plotted in function of the pair-distance, r, as determined by GNOM. (E) Cartoon representation of the full-atom model of the ternary TSLP complex (left) generated by the AllosMod-FoxS server on basis of the determined X-ray structure that resulted in the best fit (χ= 2.12) between the theoretical and observed scattering profile up to a q-value of 0.25 $Å^{-1}$ (right).

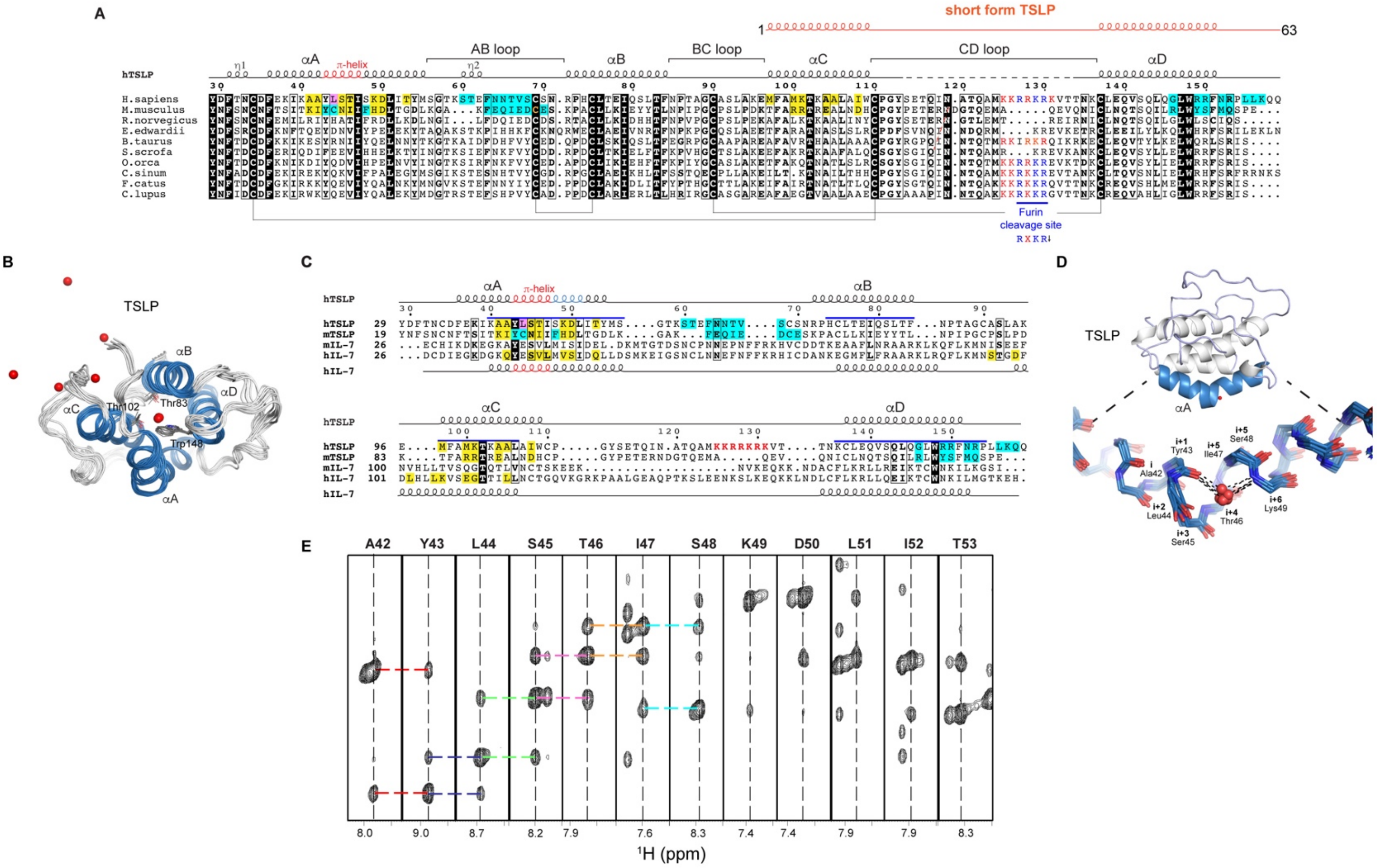

Supplementary Figure 3

(A) Sequence comparison between selected TSLP orthologs. Residue numbering corresponds to human TSLP. Strictly conserved residues are colored white on a black background. Semi-conserved residues are boxed and shown in black bold. Residues that interact with TSLPR are highlighted in cyan and residues that interact with IL-7Rα in yellow. Human TSLP-Leu45 interacts with both TSLPR and IL-7Rα and is highlighted in pink. The secondary structure of human TSLP as seen in the TSLP:TSLPR:IL-7Rα structure is shown on top. Disulfide pairs are indicated by black lines. Arg/Lys residues in the basic stretch in the CD-loop are colored red, with residues constituting the furin cleavage site colored blue. The possible secondary structure of short form TSLP is shown on top. (B) MD-simulations on TSLP from which the central water molecule was deleted in the starting structure show that a water molecule (red spheres) from bulk solvent can enter through a water channel that is located between helices B and C. Shown is the path that the water molecule is following. (C) Structure-based sequence comparison between human (h) and mouse (m) TSLP and IL-7 cytokines, based on the structural alignment of the X-ray structures of human TSLP and IL-7 shown in Figure 2D. Segments included in the structural alignment are indicated by blue bars. Strictly conserved residues are colored white on a black background. Semi-conserved residues are boxed and shown in black bold. TSLPR-interacting residues of hTSLP and mTSLP are highlighted in cyan. IL-7Rα-interacting residues of hTSLP, mTSLP and hIL-7 are highlighted in yellow. The basic stretch of hTSLP, located in the CD loop is colored in red. (D) During the 5 x 250 ns MD-simulations for TSLP, an inserted water molecule (red spheres) at the π-helical turn in helix A was found in 19% of all frames. Dashed lines indicated hydrogen bounds (E) NOESY strips through the amide resonances of the residues in helix A. Cross peaks are strongest around the region where the kink is identified in the crystal structure.

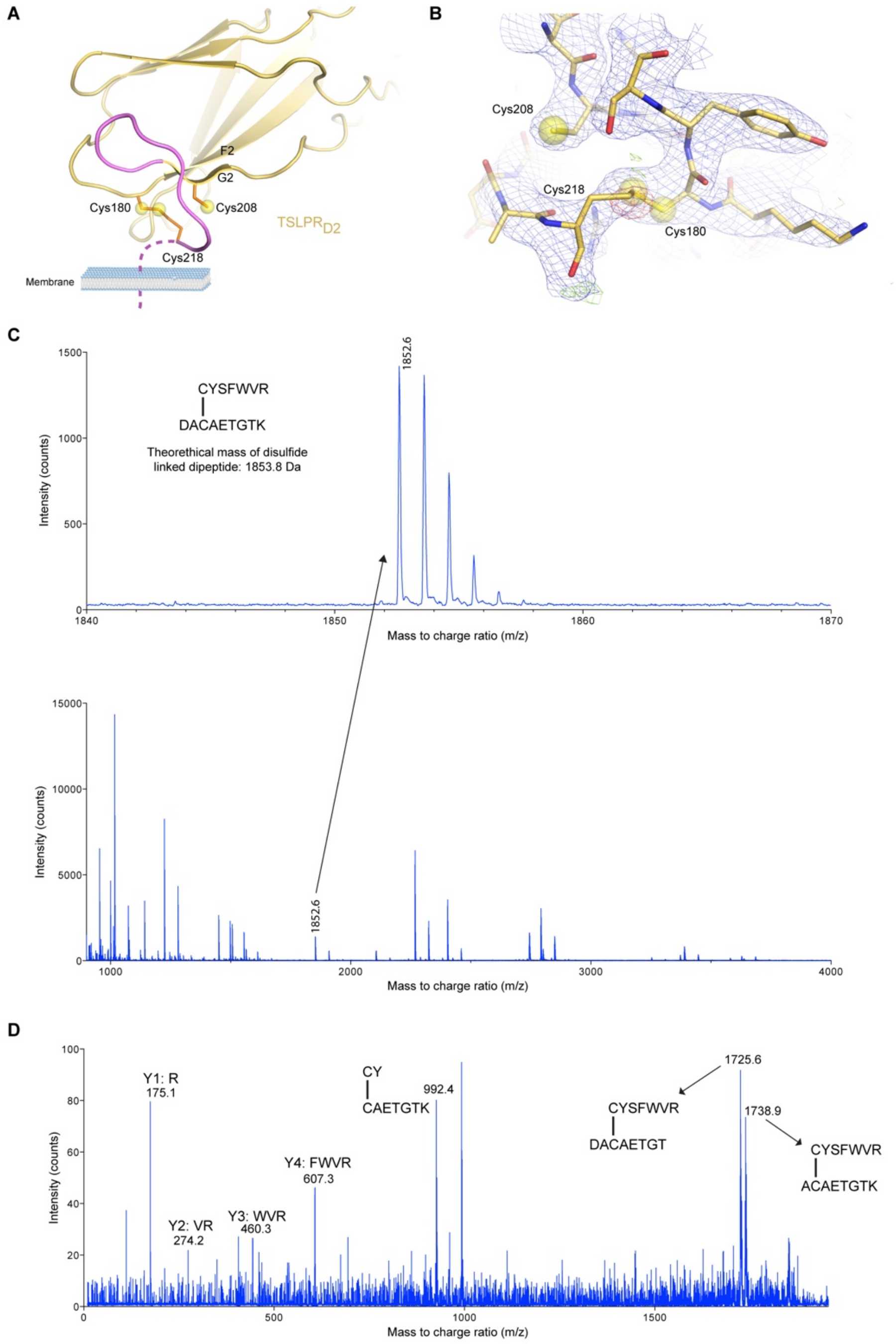

Supplementary Figure 4

Triangle of cysteine residues at membrane-proximal region of TSLPR (A) Cartoon representation of the membrane-proximal TSLPR$_{D2}$ domain as seen in the TSLP:TSLPR:IL-7Rα X-ray structure with a focus on the loop-region extending from strand G2 (purple) that connects to the transmembrane helix. The Cys180-Cys218 disulfide and residue Cys208 are shown as ball-and-sticks. (B) Quality of the electron density maps around the Cys180-Cys218 disulfide. Sigma$_A$-weighted 2mF$_O$-DF$_C$ electron density map contoured at an r.s.m.d. of 1 (blue mesh) overlayed with a Sigma$_A$-weighted mF$_O$-DF$_C$ difference map contoured at ±3 r.m.s.d., with negative difference peaks in red and positive difference peaks in green. The figure was rendered in PyMOL[1] using a carve radius of 2.0 Å. (C) MALDI-MS peptide spectrum of soluble TSLPR ectodomain contains a peak corresponding to the Cys180-Cys218 disulfide-linked dipeptide as observed in the X-ray structure. Bottom: full MALDI-MS spectrum. Top: detail of the region containing the disulfide-linked peptide (D) Resulting MALDI-MS/MS spectrum with peptide fragments originating from the disulfide-linked peptide indicated.

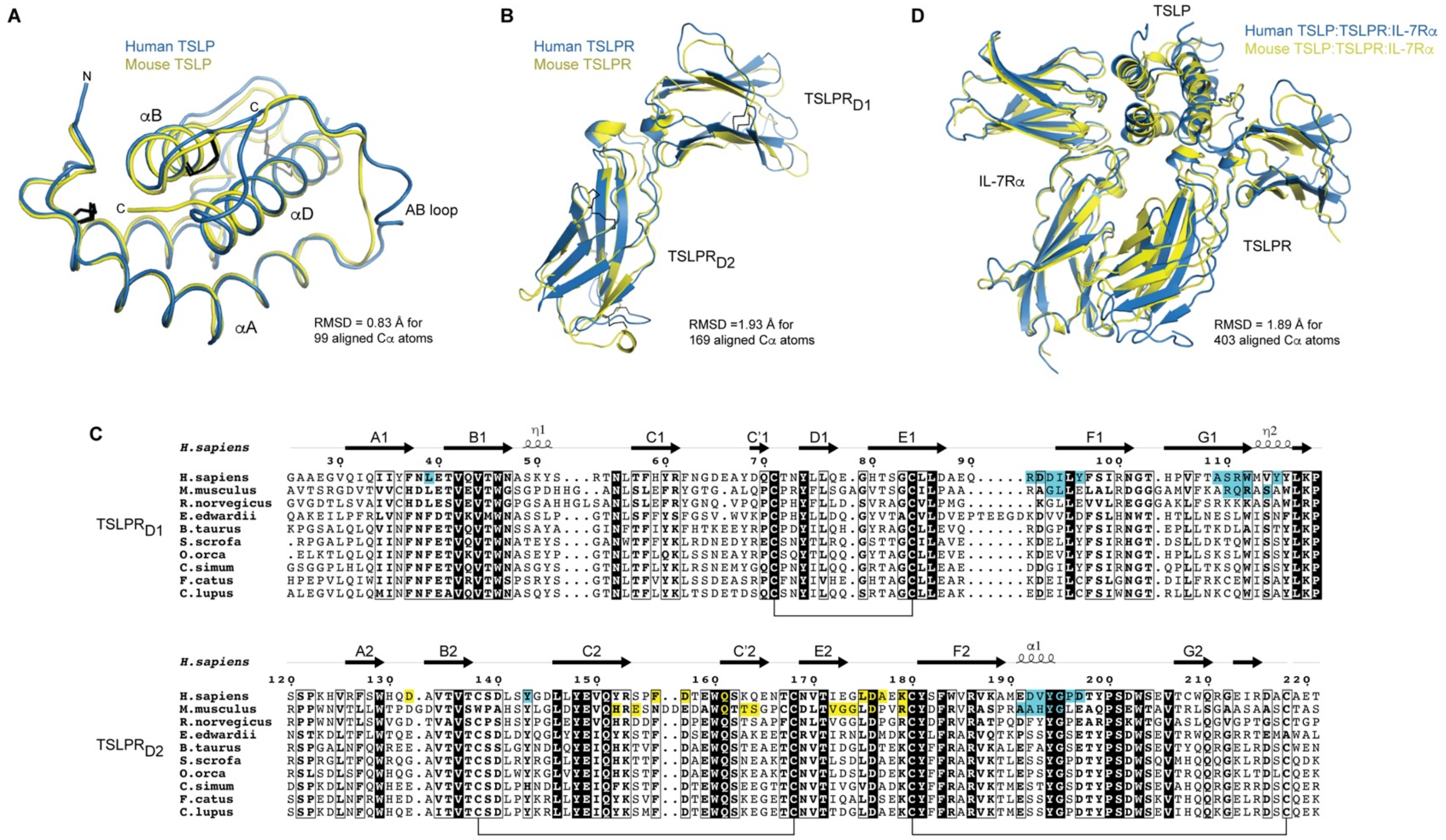

Supplementary Figure 5

(A) Structural alignment of human (blue, pdb 5J11) and mouse (yellow, pdb 4NN5) TSLP. Helices αA to αD, and the N-and C-termini are labeled. (B) Structural alignment of human (blue, pdb 5J11) and mouse (yellow, pdb 4NN5) TSLPR. Disulfide bridges are shown as black lines. Disulfide bridges are shown as black lines. (C) Sequence comparison between selected TSLPR orthologs. Residue numbering corresponds to human TSLPR. Strictly conserved residues are colored white on a black background. Semi-conserved residues are boxed and shown in black bold. Human and mouse TSLPR residues that interact with TSLP are highlighted in cyan and residues that interact with IL-7Rα in yellow. The secondary structure of human TSLPR is shown on top. Disulfide pairs are indicated by black lines. (D) Overall structural alignment between human (blue, pdb 5J11) and mouse (orange, pdb 4NN5) ternary TSLP complexes.

Structures were superimposed with Chimera[2]. Sequence alignments of TSLP, TSLPR and IL-7Rα from different species were created using Clustal Omega[3] and formatted with ESPript [4]. Secondary structures elements of crystallographic structures were assigned with DSSP[5,6]. The electrostatic potential at solvent accessible surface was calculated using APBS[7,8] and PDB2PQR[9]. Panels a-c containing structural models were prepared in PyMOL[1].

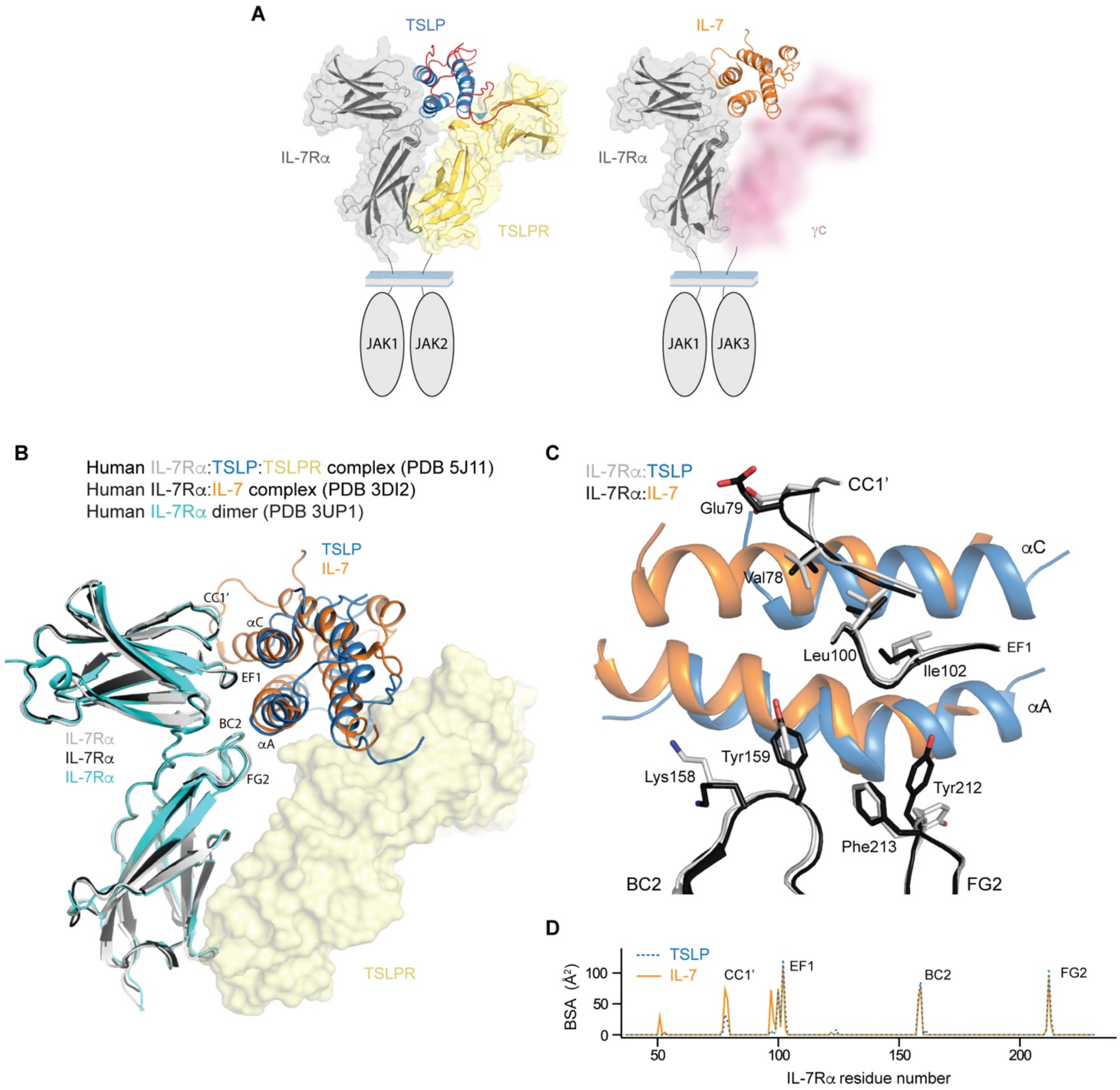

Supplementary Figure 6

Structural basis for the molecular degeneracy of shared IL-7Rα. (A) Cartoon representation illustrating the dual role of IL-7Rα at the cell surface based on the ternary TSLP:TSLPR:IL-7Rα structure (left) and the binary IL-7:IL-7Rα structure (pdb 3DI2, right). The common gamma chain (cγ) is shown as a pink blur. (B) Structural comparison of human IL-7Rα structures as seen in the ternary TSLP:TSLPR:IL-7Rα complex (grey), the binary IL-7:IL-7Rα complex (black, pdb 3DI2) and the IL-7Rα:IL-7Rα structure (cyan, pdb 3UP1). Loop segments of IL-7Rα that are involved in cytokine-binding and cytokine helices αA and αC are labeled. TSLP (blue) and IL-7 (orange) are shown as cartoons. TSLPR is shown in surface mode (yellow) (C) Detail of the IL-7Rα cytokine-receptor interfaces based on the alignment of IL-7Rα. IL-7Rα residues that bind TSLP (blue) and IL-7 (orange) are shown as sticks. (D) Plot of the buried surface area (BSA) per IL-7Rα residue upon binding TSLP (dashed blue line) or IL-7 (orange line).

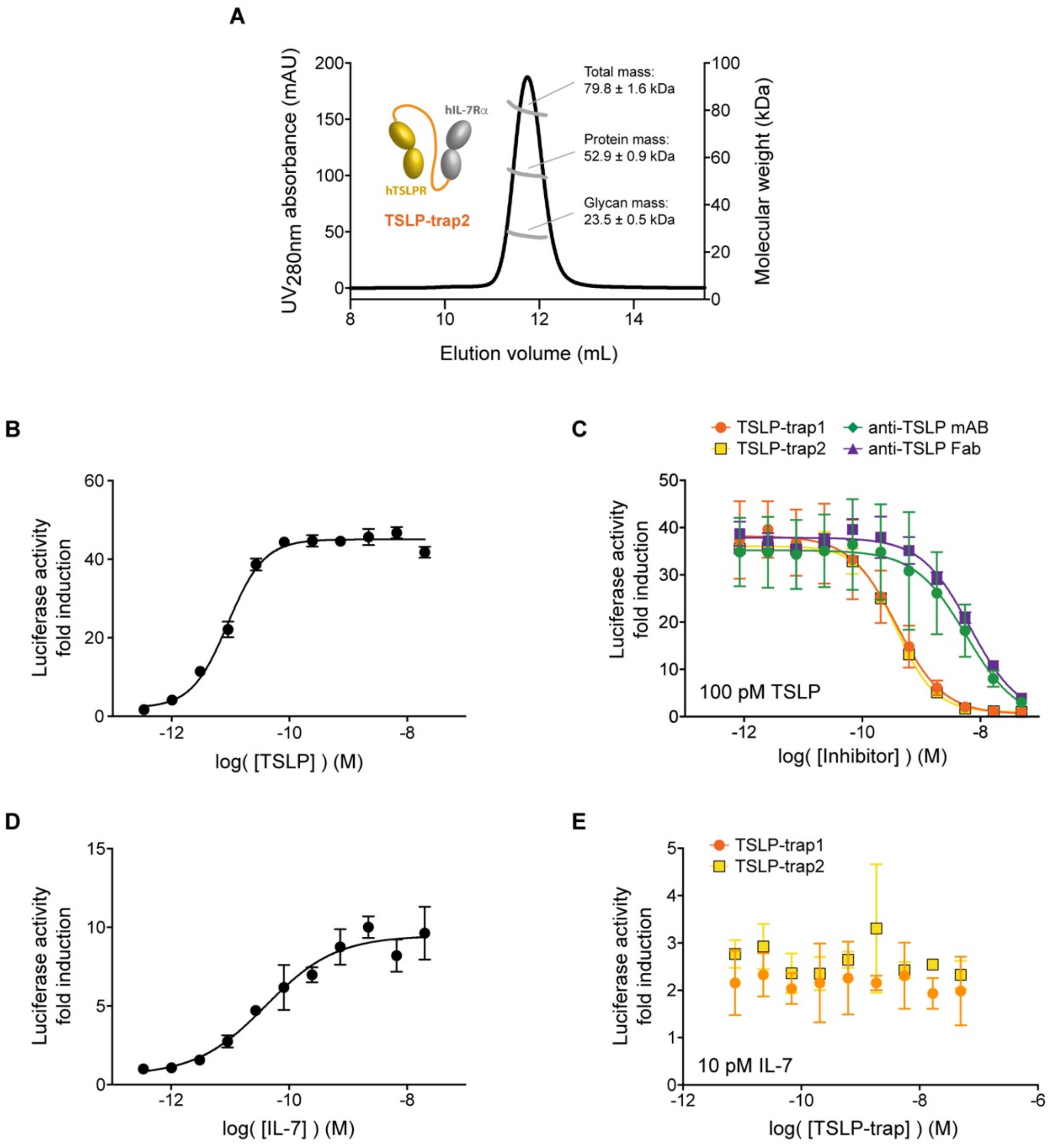

Supplementary Figure 7

(A) SEC elution profile of TSLP-trap2 plotted as the UV absorbance at 280 nm (left Y-axis) in function of elution time. The total, protein and glycan molecular weight (right Y-axis) as determined by MALLS are reported as the number average molecular mass and standard deviation. (B) Dose-response curve of TSLP-induced STAT5-activity in HEK293T cells transfected with TSLPR and IL-7Rα. The determined EC_{50}-value was 9 pM. (C) STAT5 activity induced by 100 pM TSLP in function of increasing concentrations of TSLP antagonists. The determined IC_{50}-values were 382 pM for TSLP-trap1; 382 pM for TSLP-trap2; 5.8 nM for AMG-157 and 6.7 nM for AMG-157_{Fab}. (D) Dose-response curve of IL-7-induced STAT5-activity in HEK293 cells transfected with IL-7Rα and the common chain receptor. The determined EC_{50}-value was 41 pM. (E) STAT5 activity induced by 10 pM IL-7 plotted in function of increasing concentrations of TSLP-traps. STAT5 activity in HEK293T cells is plotted as the luciferase activity fold induction; cellular assays were carried out in triplicate; data shown are averages, and error bars were calculated as s.e.m.

A

anti-TSLP mAb light chain

```
         10         20         30         40         50         60
ETGSYVLTQP PSVSVAPGQT ARITCGGNNL GSKSVHWYQQ KPGQAPVLVV YDDSDRPSWI

         70         80         90        100        110        120
PERFSGSNSG NTATLTISRG EAGDEADYYC QVWDSSSDHV VFGGGTKLTV LGQPKAAPSV

        130        140        150        160        170        180
TLFPPSSEEL QANKATLVCL ISDFYPGAVT VAWKADSSPV KAGVETTTPS KQSNNKYAAS

        190        200        210
SYLSLTPEQW KSHRSYSCQV THEGSTVEKT VAPTECS
```

anti-TSLP mAb heavy chain

```
         10         20         30         40         50         60
ETGQMQLVES GGGVVQPGRS LRLSCAASGF TFRTYGMHWV RQAPGKGLEW VAVIWYDGSN

         70         80         90        100        110        120
KHYADSVKGR FTITRDNSKN TLNLQMNSLR AEDTAVYYCA RAPQWELVHE AFDIWGQGTM

        130        140        150        160        170        180
VTVSSASTKG PSVFPLAPCS RSTSESTAAL GCLVKDYFPE PVTVSWNSGA LTSGVHTFPA

        190        200        210        220        230        240
VLQSSGLYSL SSVVTVPSSN FGTQTYTCNV DHKPSNTKVD KTVERKCCVE CPPCPAPPVA

        250        260        270        280        290        300
GPSVFLFPPK PKDTLMISRT PEVTCVVVDV SHEDPEVQFN WYVDGVEVHN AKTKPREEQF

        310        320        330        340        350        360
NSTFRVVSVL TVVHQDWLNG KEYKCKVSNK GLPAPIEKTI SKTKGQPREP QVYTLPPSRE

        370        380        390        400        410        420
EMTKNQVSLT CLVKGFYPSD IAVEWESNGQ PENNYKTTPP MLDSDGSFFL YSKLTVDKSR

        430        440        450        460
WQQGNVFSCS VMHEALHNHY TQKSLSLSPG KGTKHHHHHH
```

B

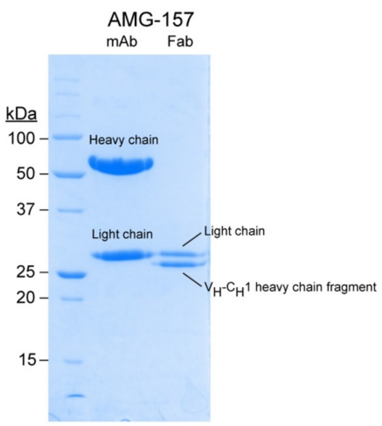

C

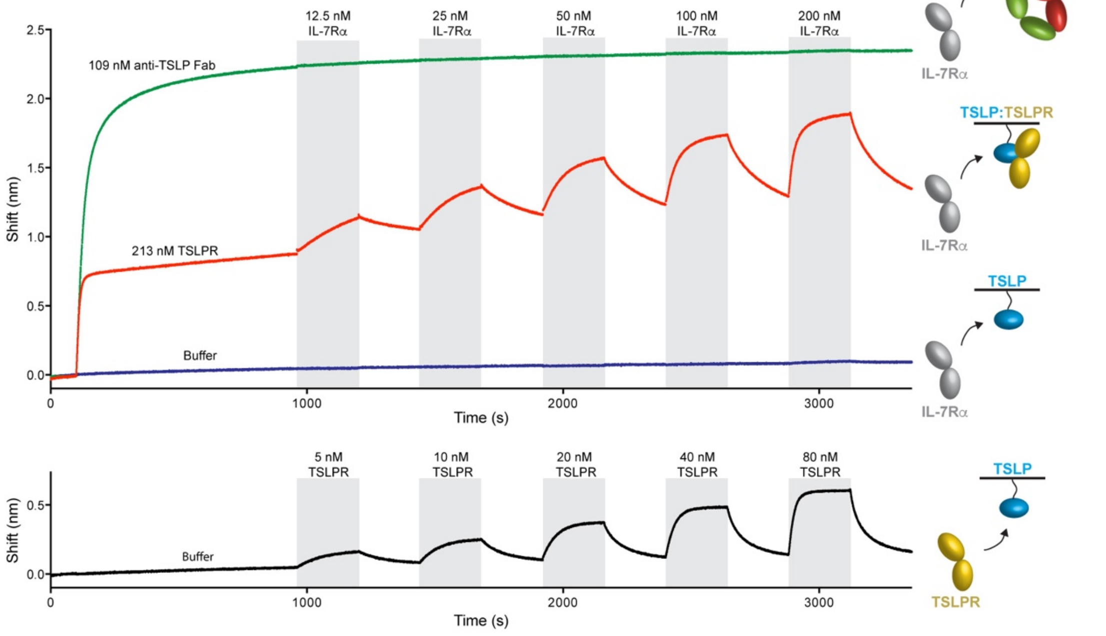

D

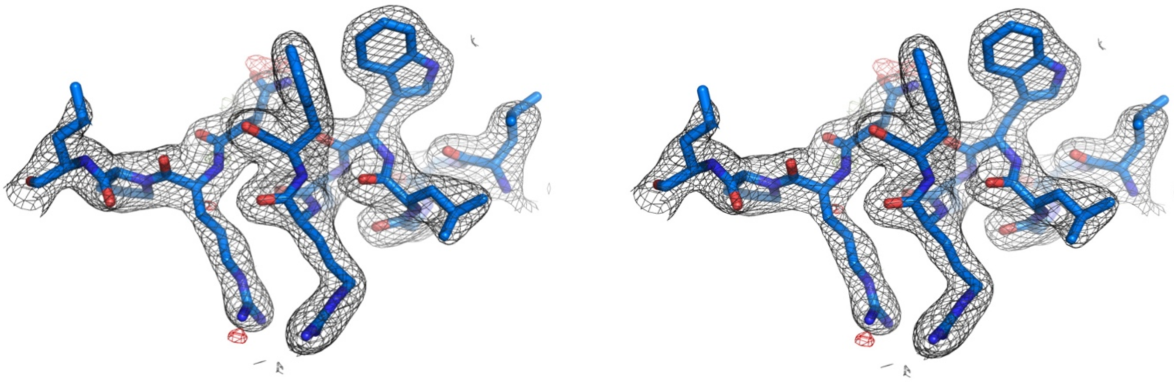

Supplementary Figure 8

Amino acid sequences for the light and heavy chain of the in-house produced anti-TSLP mAb corresponding to AMG-157 (Tezepelumab). The N-terminal residues, Glu-Thr-Gly, for both the light and the heavy chain, result from cleavage of the signal peptide encoded in the pHLsec vector. The heavy chain carries a hexahistidine-tag at the C-terminus to facilitate purification. Complementary determining regions are highlighted in blue. AMG-157 mAb residues interacting with TSLP as observed in the TSLP:AMG-157$_{Fab}$ X-ray structure are highlighted in green. (B) Coomassie-stained reducing SDS-PAGE gel of purified AMG-157 mAb and it derived Fab fragment. The theoretical protein molecular weights are 50.9 kDa for the IgG2 heavy chain, 23.1 kDa for the lambda light chain and 25,6 kDa for the V_H-C_H1 heavy chain fragment. Molecular weights of protein standards are indicated. (C) Single-cycle BLI data traces are plotted as the observed spectral nanometer shift in function of time for the interaction of IL-7Rα with TSLP (blue), IL-7Rα with TSLP:TSLPR complex (red) and IL-7Rα with TSLP:AMG-157$_{Fab}$ complex (green). In a parallel experiment (bottom), the binding profile of the TSLPR:TSLP interaction is shown (black trace). To generate the biosensing surfaces 1.5 nm of biotinylated TSLP was loaded on streptavidin-coated sensor tips; 213 nM of TSLPR was used to generated the TSLP:TSLPR surface and included in subsequent IL-7Rα samples and buffer wells; 109 nM of AMG-157$_{Fab}$ was used to generate the TSLP:AMG-157$_{Fab}$ surface and included in subsequent IL-7Rα samples and buffer wells. (D) Stereo-view of the final Sigma-A weighted 2mF_o-DF_c and mF_o-DF_c difference electron density maps for the TSLP:Fab complex around TSLP residues 144 to 155. The 2mF_o-DF_c map (grey) is contoured at 1 r.m.s.d. and the mF_o-DF_c map at ±3 r.m.s.d., and were rendered in PyMOL[1] using a carve radius of 1.6 Å.

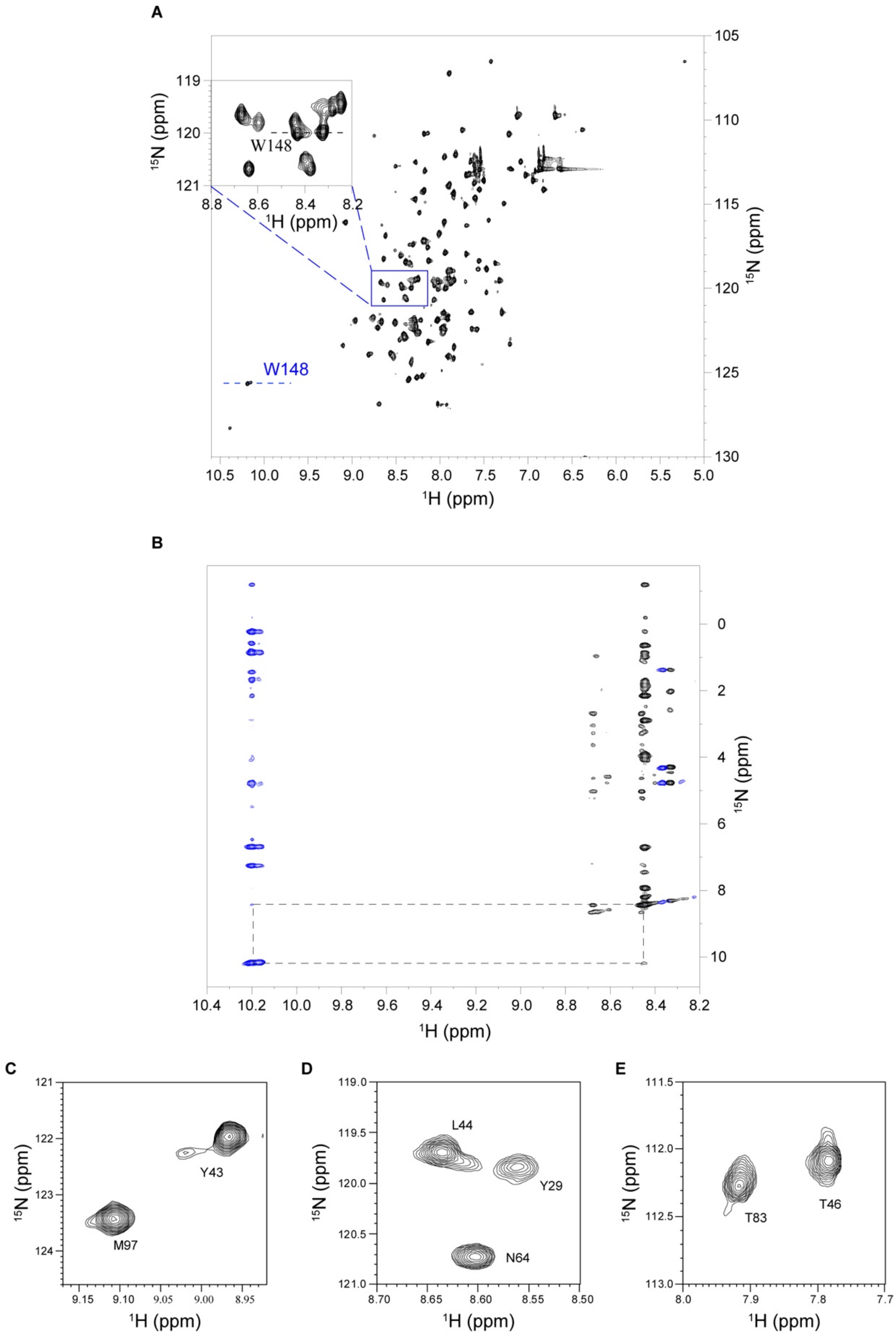

Supplementary Figure 9

Conformational heterogeneity of TSLP at the π-helical in turn helix A. (A,B) Assignment of the Trp side chain to the Trp148 residue. NOE strips through the side chain (blue line) and backbone (black) amide resonance of Trp148 (panel A) display the NOE contact connecting both protons (panel B). (C-E) Structural heterogeneity in the π-helical turn of helix αA extends beyond Ser45, as witnessed by the minor peaks in the ^{1}H, ^{15}N HSQC spectrum at 900MHz for Tyr43 (panel E), Leu44 (panel F) and Thr46 (panel G).

Fig. 1F

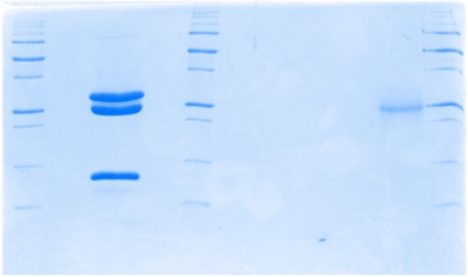

Fig. 5B
Supplementary Fig. 1A and 8B

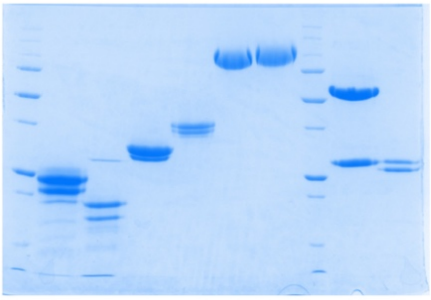

Supplementary Figure 10
Uncropped Coomassie-stained SDS-PAGE gels used in main display items Figure 1F, Figure 5B, Supplementary Figure 1A, and Supplementary Figure 8B.

Supplementary Table 1: SAXS data-collection and scattering-derived parameters

**Data collection parameters**	
Instrument	Swing beam line (SOLEIL synchrotron)
Experimental setup	SEC-SAXS [10]
Beam geometry	0.45 x 0.02 mm^2
Wavelength (Å)	1.03 Å
q range ($Å^{-1}$)	0 – 0.6 Å
SEC flow rate (mL/min)	200
SEC column	Agilent Bio SEC-3 with 300 Å pore size
Exposure time per frame	1 s
Acquisition rate (frame/s)	1 frame per 2 seconds
Temperature (K)	288
**Structural parameters**	
I_0 (cm^{-1}) [from P(r)]	0.053
R_g (Å) [from P(r)]	31.35
I_0 (cm^{-1}) [from Guinier]	0.053
R_g (Å) [from Guinier]	31.24
D_{max} (Å) [from P(r)]	103.19
Excluded volume ($Å^3$)	98021
**Molecular weight (MW) determination**	
#MW (kDa) from $I_{0,exp}$	58.7
MW (kDa) from ScÅtter	56.0
‡MW (kDa) from Saxs-MoW2	76.2
MW(kDa) from Porod volume	61.3
$Calculated MW (kDa) from sequence	64,3
Determined MW (kDa) by MALLS	64,4
**Software employed**	
Primary data reduction	Foxtrot
Guinier analysis	Foxtrot, AutoRg
P(r) calculation	GNOM 5
Particle exclude volume	DATPOROD
Molecular weight estimation	ScÅtter[11], SAXS-MoW2[12], DATPOROD[13]
Model generation and fitting	AllosMod-FoXS server[14]

#Molecular mass was determined from $I_{0,exp}$ using water scattering as a reference

‡with q_{max}= 0.26 $Å^{-1}$ (= 8/R_g)

$Without glycosylation. It is expected that following EndoH-treatment of the $TSLPR^{N47Q}$ two to three GlcNAc residues are still present, increasing the mass with about 600 Da.

Supplementary Table 2. **Interactions at the TSLP:TSLPR, TSLP:IL-7Rα and IL-7Rα:TSLPR interfaces**

Site 1. TSLP:TSLPR interface *

Potential H-bonds and salt bridges

TSLP	Distance (Å)	TSLPR
Arg 153 Nε	3.55	Asp 92 Oδ1
Arg 153 Nε	2.7	Asp 92 Oδ2
Arg 153 Nη1	2.94	Val 193 O
Arg 153 Nη2	3.09	Asp 92 Oδ1
Arg 153 Nη2	3.38	Asp 92 Oδ2
Arg 149 Nη1	3.11	Tyr 115 Oη
Arg 150 Nη1	2.72	Asp 92 Oδ2
Gln 158 Nε2	2.87	Asp 92 O
Asn 65 N	2.99	Ser 110 O
Gln 159 N	2.97	Asp 93 Oδ2
Ser 60 Oγ	2.97	Asp 197 Oδ2
Lys 157 O	2.77	Asp 92 N
Thr 66 O	3.02	Tyr 96 Oη
Asn 64 Oδ1	2.85	Ser 110 N
Asn 65 O	2.75	Ser 110 Oγ
Phe 63 O	2.89	Arg 111 Nε
Thr 61 O	2.72	Arg 111 Nη1
Asn 64 Oδ1	3.37	Arg 111 Nη2
Asn 65 Oδ1	3.49	Trp 112 N

Van der Waals contacts

TSLP	TSLPR
Leu 44	Asp 192
Thr 61	Asp 197
Phe 63	Arg 111
Asn 64	Ala 109
Asn 65	Ile 94, Arg 111, Trp 112
Val 67	Ile 94
Ser 68	Trp 112
Gly 146	Tyr 115, Trp 112
Arg 149	Tyr115, Asp192, Val193, Gly195, Pro196
Arg 150	Trp 112
Asn 152	Val 193
Arg 153	Val 114, Val 193, Trp 112, Tyr 194
Leu 156	Asp 92, Leu 39, Tyr 143
Gln 158	Asp 93, Arg 91

Bridging water molecules

Water molecule	hTSLP	hTSLPR
H2O 20	Asn152 [O]	Asp145 [Oδ1]
H2O 10	Val67 [O]	Trp112 [Nε1]
H2O 23	Asn65 [Nδ2]	Tyr115 [Oη]

Site 2. TSLP:IL-7Rα interface*

Potential H-bonds and salt bridges

TSLP	Distance (Å)	IL-7Rα
Lys 49 Nζ	3.02	Tyr 159 O
Lys 101 Nζ	3.41	Tyr 159 Oη
Asp 50 Oδ1	2.71	Tyr 159 Oη
Ser 45 Oγ	2.95	Tyr 212 O
Thr 53 Oγ1	3.33	Lys 158 Nζ

Bridging water molecules

Water molecule	TSLP	IL-7R
H2O 33	Thr46 Oγ1	Leu101 O

Van der Waals contacts

TSLP	IL-7Rα
Ala 41	Tyr 212
Ala 42	Ile 102
Leu 44	Tyr 212
Ser 45	Tyr 212 Phe 213
Lys 49	Lys 158, Tyr 159
Thr 53	Lys 158
Met 100	Val 78
Met 97	Val 78, Phe 99
Lys 101	Leu 100, Ile 102
Ala 104	Leu 100, Ile 102
Ala 105	Ile 102
Ile 108	Ile 102, Gly 103

Site 3. IL-7Rα:TSLPR interface*

Potential salt bridges and hydrogen bonds

IL-7Rα	Distance (Å)	TSLPR
Arg 140 Nη1	3.02	Phe 156 O
Arg 140 Nη1	2.95	Asp 157 Oδ1
Arg 140 Nη2	3.59	Asp 157 Oδ1
Asp 145 Oδ2	2.76	Lys 179 Nζ
Arg 193 Nη2	3.73	Asp 132 Oδ2
Arg 193 Nη1	3.21	Asp 132 Oδ2
Arg 193 Nη2	2.70	Asp 176 Oδ1
Arg 193 Nη2	2.75	Asp 132 Oδ1
Arg 193 Nε	3.65	Asp 176 Oδ1
Arg 193 Nη2	2.75	Asp 132 Oδ1

Van der Waals contacts

IL-7Rα	TSLPR
Leu 191	Asp 176, Leu 175
Ala 143	Lys 179, Phe 156
Gly 142	Phe 156
Arg 140	Phe 156
Arg 193	Ala 177
Lys 187	Gln 161

*Protein-protein interaction interfaces were analyzed using PDBePISA [15] and the "Find Clashes/Contacts" module in Chimera[2]. The volume of the internal cavity in TSLP was calculated with the CASTp server[16], using a probe radius of 1.1 Å.

Supplementary Table 3. Cellular structure-function studies with TSLP, TSLPR and IL7Rα mutants

TSLP mutants Sites I + II	STAT5 activation		95% Confidence Intervals		R squared
	Max. fold induction	EC50 (M)	Max. fold induction	EC50 (M)	
Series 1					
TSLP-WT	15,2	1,5E-13	14,8 to 15,7	8,7E-14 to 2,4E-13	0,98
TSLP-S45K	10,8	1,1E-13	8,2 to 13,4	8,9E-15 to 1,4E-12	0,31
TSLP-T46E	13,4	3,4E-13	12,7 to 14,2	2,3E-13 to 5,1E-13	0,95
TSLP-S45K/T46Q	13,0	2,7E-13	11,8 to 14,2	1,3E-13 to 5,6E-13	0,83
TSLP-K49S	13,8	2,0E-13	13,2 to 14,5	1,4E-13 to 2,9E-13	0,94
TSLP-D50A	11,5	2,6E-13	9,6 to 13,3	1,0E-13 to 6,5E-13	0,84
TSLP-M97S	15,8	3,6E-14	15,2 to 16,5	1,8E-14 to 7,15E-14	0,94
TSLP-M100S	12,2	2,3E-13	11,0 to 13,5	6,2E-14 to 8,4E-13	0,80
TSLP-K101S	13,0	4,0E-13	12,3 to 13,7	2,7E-13 to 5,9E -13	0,95
TSLP-K101E	14,6	4,5E-13	12,9 to 16,4	9,1E-14 to 2,2E -12	0,84
TSLP-A104Q	14,9	7,1E-14	14,2 to 15,6	4,8E-14 to 1,0E -13	0,94
TSLP-I108S	14,5	6,8E-14	13,2 to 15,8	2,3E-14 to 2,0E -13	0,85
TSLP-W109A	16,8	1,3E-13	15,9 to 17,6	6,2E-14 to 2,7E-13	0,94
TSLP-E62A	11,7	1,2E-13	10,9 to 12,5	4,4E-14 to 3,5E-13	0,83
TSLP-R149S	15,2	2,8E-12	14,4 to 16,0	2,0E-12 to 4,0E-12	0,98
TSLP-R149S+R150S	11,2	1,0E-10	9,0 to 13,3	5,9E-11 to 1,8E-10	0,90
TSLP-R150S	12,6	9,5E-12	11,8 to 13,3	7,2E-12 to 1,3E-11	0,98
TSLP-R153S	13,6	1,9E-11	11,3 to 16,0	9,3E-12 to 3,9E-11	0,91
Series 2					
TSLP-WT	13,8	1,1E-13	13,4 to 14,2	8,5E-14 to 1,4E-13	0,97
TSLP-T46K	14,9	2,0E-13	13,6 to 16,3	1,1E-13 to 3,7E-13	0,83
TSLP-S45R/T46R	14,8	5,3E-12	14,1 to 15,5	4,2E-12 to 6,7E-12	0,98
TSLP-A42R	15,0	2,0E-13	14,4 to 15,5	1,5E-13 to 2,6E-13	0,97
TSLP-A42E	14,8	3,8E-13	13,6 to 16,2	1,7E-13 to 8,1E-13	0,88
TSLP-A42D	15,2	2,8E-13	14,6 to 15,8	2,1E-13 to 3,8E-13	0,97
TSLP-A104E	17,7	9,8E-14	17,2 to 18,2	7,5E-14 to 1,3E-13	0,98
TSLP-A104D	14,5	5,5E-13	13,6 to 15,4	3,5E-13 to 8,6E-13	0,95
TSLP-A104R	13,6	1,4E-13	13,6 to 14,1	1,0E-13 to 1,8E-13	0,97

	Competitive binding		95% Confidence Intervals		R squared
	Max. luciferase activity (cps)	IC50 (M)	Max. luciferase activity (cps)	IC50 (M)	
TSLP-WT	**71231**	3,2E-10	59732 to 82730	2,0E-10 to 5,4E-10	0,93
TSLP-S45R/T46R	**83601**	7,2E-10	73057 to 94145	4,3E-10 to 1,2E-9	0,93

TSLPR/IL-7Rα mutants Sites I + II	Max. fold induction	EC50 (M)	95% Confidence Intervals		R squared	FACS expression*		Remarks
			Max. fold induction	EC50 (M)		TSLPR	IL-7Rα	
TSLPR-WT + IL-7Rα-WT	24,5	8,5E-11	23,6 to 25,5	7,3E-11 to 9,9E-11	0,99	2832	440	
Mock						516	154	
TSLPR-W112A	~ 1172	~ 2,8E-7	(Very wide)	(Very wide)	0,98	3756	624	*Ambiguous*
TSLPR-R111A	35,9	1,7E-10	33,0 to 38,8	1,3E-10 to 2,3E-10	0,95	3308	750	
TSLPR-W112R						3216	687	*Not converged*
TSLPR-Y115A	65,9	1,9E-10	63,4 to 68,4	1,7E-10 to 2,2E-10	0,99	3319	651	
TSLPR-W112A/Y115A	13,7	5,0E-09	-21,4 to 48,9	2,2E-10 to 1,1E-7	0,93	5436	733	***
TSLPR-D92A	~ 648,7	~ 8,7E-8	(Very wide)	(Very wide)	0,95	4081	696	*Ambiguous*
TSLPR-D93A	35,0	2,2E-10	32,0 to 38,1	1,6E-10 to 3,0E-10	0,97	3970	527	
TSLPR-D192A	37,1	1,3E-10	35,3 to 38,9	1,1E-10 to 1,6E-10	0,99	2837	598	
IL-7Rα-L100S/I102S	27,9	4,7E-10	24,4 to 31,4	3,3E-10 to 6,8E-10	0,92	3332	545	
IL-7Rα-L100S	36,6	1,3E-10	35,3 to 37,8	1,1E-10 to 1,4E-10	0,99	3621	576	
IL-7Rα-L101S	28,8	1,6E-10	25,5 to 32,0	1,1E-10 to 2,4E-10	0,91	4524	637	
IL-7Rα-I102S	35,0	2,2E-10	33,5 to 36,5	1,9E-10 to 2,5E-10	0,99	4681	638	
IL-7Rα-Y159S	33,8	1,8E-10	32,3 to 35,3	1,5E-10 to 2,1E-10	0,98	4045	695	
IL-7Rα-K158S	22,0	5,4E-11	20,4 to 23,6	4,0E-11 to 7,4E-11	0,95	3030	417	
IL-7Rα-K158S/Y159S	26,8	1,5E-10	24,5 to 29,0	1,1E-10 to 2,0E-10	0,94	3067	547	

TSLPR mutants Site III	Max. fold induction	EC50 (M)	95% Confidence Intervals		R squared	FACS expression*	TSLP-SEAP binding (% of WT)	Remarks
			Max. fold induction	EC50 (M)		TSLPR		
Series 1								
TSLPR-WT	14,6	1,1E-11	13,6 to 15,5	7,5E-12 to 1,6E-11	0,96	2050	100	
Mock						93	1	
TSLPR-D157A/E159A/Q161A	12,2	1,0E-10	11,7 to 12,7	8,6E-11 to 1,2E-10	0,99	1939	93	
TSLPR-D157A/E159A	14,0	8,5E-11	13,0 to 15,1	6,2E-11 to 1,2E-10	0,97	2891	78	
TSLPR-F156A/D157A/E159A	17,2	4,0E-10	15,9 to 18,6	3,2E-10 to 4,9E-10	0,99	2329	90	
TSLPR-D176A/E178A/K179A	12,5	4,9E-11	12,0 to 13,0	4,0E-11 to 5,9E-11	0,99	1959	94	
Series 2								
TSLPR-WT	14,9	1,2E-11	13,5 to 16,3	7,6E-12 to 1,8E-11	0,96	2050	100	
Mock						93	1	
TSLPR-F156A	14,4	2,2E-11	12,8 to 16,1	1,4E-11 to 3,6E-11	0,93	1445	107	
TSLPR-D157A	1,0	~ 7,5E-11	1,0 to 1,1		0,23	91	1	*Ambiguous*
TSLPR-E159A	14,5	1,3E-11	13,3 to 15,7	9,3E-12 to 1,9E-11	0,96	1851	96	
TSLPR-D176A	14,4	2,1E-11	13,3 to 15,5	1,5E-11 to 2,9E-11	0,96	2681	93	
TSLPR-E178A	15,0	1,3E-11	14,1 to 15,9	9,7E-12 to 1,6E-11	0,97	2481	90	
TSLPR-K179A	13,3	1,9E-11	12,6 to 14,0	1,5E-11 to 2,3E-11	0,98	2742	79	

***The TSLPR-W112A/Y115A TSLPR mutant has a drastically increased EC50 value. For this mutant, data points for TSLP concentrations above its EC50 value are missing, and values inferred from curve fitting are less reliable.

Supplementary Table 4. **Interactions at the TSLP:AMG-157$_{Fab}$ interface**

TSLP: AMG-157$_{Fab}$ interface*

Potential H-bonds and salt bridges

TSLP	Distance (Å)	AMG-157$_{Fab}$
Asn 65 O	2.78	Arg 101 Nη2
Thr 66 O	2.73	Thr 31 N
Ser 68 N	3.20	Thr 34 Oγ1
Val 67 N	3.55	Tyr 35 Oη
Ser 70 O	2.77	Glu 106 N
Asn 71 Nδ2	3.30	Tyr 56 Oη
Arg 72 Nη1	3.93	Glu 106 Oε1
Glu 78 Oε1	3.76	Arg 33 Nη1
Glu 78 Oε2	3.18	Arg 33 Nη1
Arg 150 Nη2	2.65	Gln 104 O
Arg 150 Nη1	3.02	Glu 110 Oε1
Arg 150 Nη2	2.86	Glu 110 Oε1
Arg 153 Nη1	3.22	Glu 110 Oε1
Arg 153 Nη2	3.50	Glu 110 Oε2
Arg 153 Nη2	3.95	Glu 110 Oε1
Arg 153 Nη1	3.68	Glu 110 Oε2

Van der Waals contacts

TSLP	AMG-157$_{Fab}$
Asn71	Trp105
	Glu106
His 74	Tyr56
	Thr34
Ser70	Tyr56
	Gln104
	Trp105
Glu78	Thr34
Arg150	Pro103
Thr66	Phe30
Ser68	Thr34
	Tyr35
Arg153	Trp105
Arg150	Trp105
Val 67	Thr31
Cys75	Trp105
Asn65	Met5
	Tyr35

Bridging water molecules

Water molecule	TSLP	AMG-157$_{Fab}$
H2O 4	Ser 70 Oγ	Gln 104 N
	Ser 68 Oγ	Thr 34 O
H2O 67	Arg 72 N	Glu 110 Oε1

*Protein-protein interaction interfaces were analyzed using PDBePISA [15] and the "Find Clashes/Contacts" module in Chimera[2].

Supplementary Table 5: PCR primers used in this study.

Primer 1	CTGCTAACCATGTTCATGCCTTCT
Primer 2	GTGCTTGGTACCTGTCTCTGC
Primer 3	CTATTTCTCCATCAGGCAGGGGACGCACCCCGTTTTC
Primer 4	GAAAACGGGGTGCGTCCCCTGCCTGATGGAGAAATAG
Primer 5	CAGTCAGAATTCAAGCTTGCCACCATG
Primer 6	CTCAAGGGTACCCTTCCGTTCCACGGTCTTGTCC
Primer 7	AAAGAATTCATGGGGCGGCTGGTTCTG
Primer 8	TTTTCTAGATTTGGACAGCTTTGGTTTGGG
Primer 9	TATGAATTCATGGGGGCCGCACGCAG
Primer 10	AAATCTAGACCACAGGGGCATGTAGTCCG
Primer 11	AGATCTAGAGGAGGGAGTGGTGGCTCTGG
Primer 12	CAGCTGCAGGCTCCCACCACTCCCTCCAG
Primer 13	AAACCTGCAGGAAAGTGGCTATGCTCAAAATG
Primer 14	ATCGATATCGTTTAAACTCAATGGTGATGGTG
Primer 15	TTTCTGCAGCAAGGAGGAGCAGCAGAAG
Primer 16	AAAGAATTCGGAAAGTGGCTATGCTCAAAATG
Primer 17	GTGGGAGTGGCACCTTCC
Primer 18	CAGTCAAAGCTTGCCACCATGGGGCGGCTGGTTCTGCTG
Primer 19	CTCAAGGGTACCATCCATCTCCCCTGAGCTATTATTG
Primer 20	CAGTCAACCGGTGAAAGTGGCTATGCTCAAAATG
Primer 21	CTCAAGGGTACCTTTGGACAGCTTTGGTTTG
Primer 22	AGTGAATTCAAGCTTGCCACCATGTTCC
Primer 23	CAGAGATCTTGGATCCGCCGCTTCCACCCTGCTGTTTCA

Supplementary Table 6: Forward (fw) and reverse (rev) PCR primers used for site-directed mutagenesis of hTSLP, hTSLPR, and hIL7-Rα.

hTSLP_S15K	fw	GAGAAGATCAAAGCCGCTTACCTGAAGACTATCAGTAAGGATCTG
hTSLP_S15K	rev	CAGATCCTTACTGATAGTCTTCAGGTAAGCGGCTTTGATCTTCTC
hTSLP_T16E	fw	GAGAAGATCAAAGCCGCTTACCTGTCTGAAATCAGTAAGGATCTG
hTSLP_T16E	rev	CAGATCCTTACTGATTTCAGACAGGTAAGCGGCTTTGATCTTCTC
hTSLP_S15K/T16Q	fw	CTGCGACTTTGAGAAGATCAAAGCCGCTTACCTGCGTCAAATCAGTAAGGATCTG
hTSLP_S15K/T16Q	rev	CAGATCCTTACTGATTTGACGCAGGTAAGCGGCTTTGATCTTCTCAAAGTCGCAG
hTSLP_K19S	fw	CCTGTCTACTATCAGTTCGGATCTGATTACCTATATGTCAGGC
hTSLP_K19S	rev	GCCTGACATATAGGTAATCAGATCCGAACTGATAGTAGACAGG
hTSLP_D20A	fw	CCTGTCTACTATCAGTAAGGCTCTGATTACCTATATGTCAGG
hTSLP_D20A	rev	CCTGACATATAGGTAATCAGAGCCTTACTGATAGTAGACAGG
hTSLP_M67S	fw	GCGCATCTCTGGCCAAGGAGTCGTTTGCCATGAAGAC
hTSLP_M67S	rev	GTCTTCATGGCAAACGACTCCTTGGCCAGAGATGCGC
hTSLP_M70S	fw	GGCCAAGGAGATGTTTGCCTCGAAGACAAAAGCAGCCC
hTSLP_M70S	rev	GGGCTGCTTTTGTCTTCGAGGCAAACATCTCCTTGGCC
hTSLP_K71S	fw	GGAGATGTTTGCCATGTCGACAAAAGCAGCCCTGGC
hTSLP_K71S	rev	GCCAGGGCTGCTTTTGTCGACATGGCAAACATCTCC
hTSLP_K71E	fw	GGAGATGTTTGCCATGGAGACAAAAGCAGCCCTGGC
hTSLP_K71E	rev	GCCAGGGCTGCTTTTGTCTCCATGGCAAACATCTCC
hTSLP_A74Q	fw	GCCATGAAGACAAAACAAGCCCTGGCTATCTGGTGTCC
hTSLP_A74Q	rev	GGACACCAGATAGCCAGGGCTTGTTTTGTCTTCATGGC
hTSLP_I78S	fw	GACAAAAGCAGCCCTGGCTAGCTGGTGTCCCGGGTACAGCG
hTSLP_I78S	rev	CGCTGTACCCGGGACACCAGCTAGCCAGGGCTGCTTTTGTC
hTSLP_W79A	fw	GCAGCCCTGGCTATCGCGTGTCCCGGGTACAGCG
hTSLP_W79A	rev	CGCTGTACCCGGGACACGCGATAGCCAGGGCTGC
hTSLP_E32A	fw	GGCACTAAAAGCACCGCGTTCAACAATACAGTGAGCTGC
hTSLP_E32A	rev	GCAGCTCACTGTATTGTTGAACGCGGTGCTTTTAGTGCC

hTSLP_R119S	fw	GCTGCAGGGACTGTGGAGCAGGTTCAATCGCCCAC
hTSLP_R119S	rev	GTGGGCGATTGAACCTGCTCCACAGTCCCTGCAGC
hTSLP_R119S/R120S	fw	CTGCAGGGACTGTGGAGCAGCTTCAATCGCCCACTGCTG
hTSLP_R119S/R120S	rev	CAGCAGTGGGCGATTGAAGCTGCTCCACAGTCCCTGCAG
hTSLP_R120S	fw	CAGGGACTGTGGAGGAGCTTCAATCGCCCACTGC
hTSLP_R120S	rev	GCAGTGGGCGATTGAAGCTCCTCCACAGTCCCTG
hTSLP_R123S	fw	GTGGAGGAGGTTCAATAGCCCACTGCTGAAACAG
hTSLP_R123S	rev	CTGTTTCAGCAGTGGGCTATTGAACCTCCTCCAC
hTSLP T46K	fw	GCCGCTTACCTGTCTAAGATCAGTAAGGATCTGATTACC
hTSLP T46K	rev	GGTAATCAGATCCTTACTGATCTTAGACAGGTAAGCGGC
hTSLP S45R/T46R	fw	GAGAAGATCAAAGCCGCTTACCTGAGAAGAATCAGTAAGGATCTGATTACC
hTSLP S45R/T46R	rev	GGTAATCAGATCCTTACTGATTCTTCTCAGGTAAGCGGCTTTGATCTTCTC
hTSLP T46R/D50R	fw	GCCGCTTACCTGTCTAGAATCAGTAAGAGACTGATTACCTATATGTCAGGCAC
hTSLP T46R/D50R	rev	GTGCCTGACATATAGGTAATCAGTCTCTTACTGATTCTAGACAGGTAAGCGGC
hTSLP A42R	fw	GCGACTTTGAGAAGATCAAAGCC**AGA**TACCTGTCTACTATCAG**TAAGG**
hTSLP A42R	rev	**CCTTA**CTGATAGTAGACAGGTA**TCT**GGCTTTGATCTTCTCAAAGTCGC
hTSLP A42E	fw	GCGACTTTGAGAAGATCAAAGCC**GAG**TACCTGTCTACTATCAG
hTSLP A42E	rev	CTGATAGTAGACAGGTA**CTC**GGCTTTGATCTTCTCAAAGTCGC
hTSLP A42D	fw	GCGACTTTGAGAAGATCAAAGCCGACTACCTGTCTACTATCAG
hTSLP A42D	rev	CTGATAGTAGACAGGTAGTCGGCTTTGATCTTCTCAAAGTCGC
hTSLP A42R/T46R	fw	GCGACTTTGAGAAGATCAAAGCCAGATACCTGTCTAGAATCAGTAAGGATCTGATTACC
hTSLP A42R/T46R	rev	GGTAATCAGATCCTTACTGATTCTAGACAGGTATCTGGCTTTGATCTTCTCAAAGTCGC
hTSLP A42R/S45E/ T46R	fw	GCGACTTTGAGAAGATCAAAGCCAGATACCTGGAGAGAATCAGTAAGGATCTGATTACC
hTSLP A42R/S45E/ T46R	rev	GGTAATCAGATCCTTACTGATTCTCTCCAGGTATCTGGCTTTGATCTTCTCAAAGTCGC
hTSLP A104E	fw	GCCATGAAGACAAAAGAGGCCCTGGCTATCTGGTGTCCC
hTSLP A104E	rev	GGGACACCAGATAGCCAGGGCCTCTTTTGTCTTCATGGC
hTSLP A104D	fw	GCCATGAAGACAAAAGACGCCCTGGCTATCTGGTGTCCC

hTSLP A104D	rev	GGGACACCAGATAGCCAGGGCGTCTTTTGTCTTCATGGC
hTSLP A104R	fw	GCCATGAAGACAAAAAGAGCCCTGGCTATCTGGTGTCCC
hTSLP A104R	rev	GGGACACCAGATAGCCAGGGCTCTTTTTGTCTTCATGGC
hTSLPR D157A/E159A/Q161A	fw	GTATCGCTCACCTTTCGCTACCGCCTGGGCAAGCAAGCAGGAAAACACTTGC
hTSLPR D157A/E159A/Q161A	rev	GCAAGTGTTTTCCTGCTTGCTTGCCCAGGCGGTAGCGAAAGGTGAGCGATAC
hTSLPR D157A+E159A	fw	GCAGTATCGCTCACCTTTCGCTACCGCCTGGCAGAGCAAGCAGG
hTSLPR D157A/E159A	rev	CCTGCTTGCTCTGCCAGGCGGTAGCGAAAGGTGAGCGATACTGC
hTSLPR F156A/D157A/E159A	fw	CGAGGTGCAGTATCGCTCACCTGCCGCTACCGCCTGGCAGAGCAAGCAGG
hTSLPR F156A/D157A/E159A	rev	CCTGCTTGCTCTGCCAGGCGGTAGCGGCAGGTGAGCGATACTGCACCTCG
hTSLPR D176A/E178A/K179A	fw	GTGACCATCGAGGGGCTGGCCGCCGCAGCCTGTTATTCTTTTTGGG
hTSLPR D176A/E178A/K179A	rev	CCCAAAAAGAATAACAGGCTGCGGCGGCCAGCCCCTCGATGGTCAC
hTSLPR F156A	fw	GCAGTATCGCTCACCTGCCGATACCGAGTGGCAGAGC
hTSLPR F156A	rev	GCTCTGCCACTCGGTATCGGCAGGTGAGCGATACTGC
hTSLPR D157A	fw	GCAGTATCGCTCACCTTTCGCTACCGAGTGGCAGAGC
hTSLPR D157A	rev	GCTCTGCCACTCGGTAGCGAAAGGTGAGCGATACTGC
hTSLPR E159A	fw	CACCTTTCGATACCGCGTGGCAGAGCAAGCAGG
hTSLPR E159A	rev	CCTGCTTGCTCTGCCACGCGGTATCGAAAGGTG
hTSLPR D176A	fw	CCATCGAGGGGCTGGCCGCCGAAAAGTGTTATTC
hTSLPR D176A	rev	GAATAACACTTTTCGGCGGCCAGCCCCTCGATGG
hTSLPR E178A	fw	CGAGGGGCTGGACGCCGCAAAGTGTTATTCTTTTTGGG
hTSLPR E178A	rev	CCCAAAAAGAATAACACTTTGCGGCGTCCAGCCCCTCG
hTSLPR K179A	fw	GGGGCTGGACGCCGAAGCGTGTTATTCTTTTTGGGTGC
hTSLPR K179A	rev	GCACCCAAAAAGAATAACACGCTTCGGCGTCCAGCCCC
hTSLPR W112A	fw	CCGTGTTTACTGCTAGCCGGGCGATGGTCTACTATCTGAAGCC
hTSLPR W112A	rev	GGCTTCAGATAGTAGACCATCGCCCGGCTAGCAGTAAACACGG

hTSLPR R111A	fw	CCGTGTTTACTGCTAGCGCGTGGATGGTCTACTATCTG
hTSLPR R111A	rev	CAGATAGTAGACCATCCACGCGCTAGCAGTAAACACGG
hTSLPR W112R	fw	GTGTTTACTGCTAGCCGGAGGATGGTCTACTATCTG
hTSLPR W112R	rev	CAGATAGTAGACCATCCTCCGGCTAGCAGTAAACAC
hTSLPR Y115A	fw	GCTAGCCGGTGGATGGTCGCCTATCTGAAGCCTAGCTCC
hTSLPR Y115A	rev	GGAGCTAGGCTTCAGATAGGCGACCATCCACCGGCTAGC
hTSLPR W112A/Y115A	fw	GTGTTTACTGCTAGCCGGGCGATGGTCGCCTATCTGAAGCCTAGC
hTSLPR W112A/Y115A	rev	GCTAGGCTTCAGATAGGCGACCATCGCCCGGCTAGCAGTAAACAC
hTSLPR D92A	fw	GATGCCGAACAGCGAGCCGATATCCTGTACTTCTC
hTSLPR D92A	rev	GAGAAGTACAGGATATCGGCTCGCTGTTCGGCATC
hTSLPR D93A	fw	GCCGAACAGCGAGACGCTATCCTGTACTTCTCC
hTSLPR D93A	rev	GGAGAAGTACAGGATAGCGTCTCGCTGTTCGGC
hTSLPR D192A	fw	GTCAAAGCTATGGAGGCCGTGTACGGGCCTGATAC
hTSLPR D192A	rev	GTATCAGGCCCGTACACGGCCTCCATAGCTTTGAC
hIL7R L100S/I102S	fw	CATTGAAACAAAGAAATTTTCGCTGAGCGGGAAGTCCAATATTTGCGTG
hIL7R L100S/I102S	rev	CACGCAAATATTGGACTTCCCGCTCAGCGAAAATTTCTTTGTTTCAATG
hIL7R L100S	fw	GGAGATCTACTTCATTGAAACAAAGAAATTTTCGCTGATCGGGAAGTCC
hIL7R L100S	rev	GGACTTCCCGATCAGCGAAAATTTCTTTGTTTCAATGAAGTAGATCTCC
hIL7R L101S	fw	CTACTTCATTGAAACAAAGAAATTTCTGTCGATCGGGAAGTCCAATATTTGCGTG
hIL7R L101S	rev	CACGCAAATATTGGACTTCCCGATCGACAGAAATTTCTTTGTTTCAATGAAGTAG
hIL7R I102S	fw	GAAACAAAGAAATTTCTGCTGTCCGGGAAGTCCAATATTTGCGTG
hIL7R I102S	rev	CACGCAAATATTGGACTTCCCGGACAGCAGAAATTTCTTTGTTTC
hIL7R Y159S	fw	CCCACCTGCAGAAGAAATCCGTGAAGGTCCTGATGC
hIL7R Y159S	rev	GCATCAGGACCTTCACGGATTTCTTCTGCAGGTGGG
hIL7R K158S	fw	CTTCCCACCTGCAGAAGTCATACGTGAAGGTCCTG
hIL7R K158S	rev	CAGGACCTTCACGTATGACTTCTGCAGGTGGGAAG
hIL7R K158S/Y159S	fw	CTTCCCACCTGCAGAAGTCATCCGTGAAGGTCCTGATGC
hIL7R K158S/Y159S	rev	GCATCAGGACCTTCACGGATGACTTCTGCAGGTGGGAAG

REFERENCES

1. Schrödinger, L. The PyMOL Molecular Graphics System, Version 1.7.
2. Pettersen, E.F. et al. UCSF Chimera--a visualization system for exploratory research and analysis. *J Comput Chem* **25**, 1605-12 (2004).
3. Sievers, F. et al. Fast, scalable generation of high-quality protein multiple sequence alignments using Clustal Omega. *Mol Syst Biol* **7**, 539 (2011).
4. Gouet, P., Robert, X. & Courcelle, E. ESPript/ENDscript: Extracting and rendering sequence and 3D information from atomic structures of proteins. *Nucleic Acids Res* **31**, 3320-3 (2003).
5. Kabsch, W. & Sander, C. Dictionary of Protein Secondary Structure - Pattern-Recognition of Hydrogen-Bonded and Geometrical Features. *Biopolymers* **22**, 2577-2637 (1983).
6. Touw, W.G. et al. A series of PDB-related databanks for everyday needs. *Nucleic Acids Research* **43**, D364-D368 (2015).
7. Lerner, M.G. & Carlson, H.A. ABPS plugin for PyMOL. *University of Michigan, Ann Arbor* (2006).
8. Baker, N.A., Sept, D., Joseph, S., Holst, M.J. & McCammon, J.A. Electrostatics of nanosystems: application to microtubules and the ribosome. *Proc Natl Acad Sci U S A* **98**, 10037-41 (2001).
9. Dolinsky, T.J. et al. PDB2PQR: expanding and upgrading automated preparation of biomolecular structures for molecular simulations. *Nucleic Acids Res* **35**, W522-5 (2007).
10. David, G. & Perez, J. Combined sampler robot and high-performance liquid chromatography: a fully automated system for biological small-angle X-ray scattering experiments at the Synchrotron SOLEIL SWING beamline. *Journal of Applied Crystallograph* **42**, 892-900 (2009).
11. Rambo, R.P. & Tainer, J.A. Accurate assessment of mass, models and resolution by small-angle scattering. *Nature* **496**, 477-481 (2013).
12. Fischer, H., Oliveira Neto, M.d., Napolitano, H., Polikarpov, I. & Craievich, A. Determination of the molecular weight of proteins in solution from a single small-angle X-ray scattering measurement on a relative scale. *Journal of Applied Crystallography* **43**, 101-109 (2010).
13. Petoukhov, M.V. et al. New developments in the ATSAS program package for small-angle scattering data analysis. *Journal of applied crystallography* **45**, 342-350 (2012).
14. Guttman, M., Weinkam, P., Sali, A. & Lee, K.K. All-atom ensemble modeling to analyze small-angle x-ray scattering of glycosylated proteins. *Structure* **21**, 321-331 (2013).
15. Krissinel, E. & Henrick, K. Inference of macromolecular assemblies from crystalline state. *J Mol Biol* **372**, 774-97 (2007).
16. Binkowski, T.A., Naghibzadeh, S. & Liang, J. CASTp: Computed Atlas of Surface Topography of proteins. *Nucleic Acids Res* **31**, 3352-5 (2003).
